# Ultra-Processed Food Availability and Sociodemographic Associated Factors in a Brazilian Municipality

**DOI:** 10.3389/fnut.2022.858089

**Published:** 2022-04-20

**Authors:** Patricia Serafim, Camila Aparecida Borges, William Cabral-Miranda, Patricia Constante Jaime

**Affiliations:** ^1^Post-Graduate Program in Nutrition and Public Health, School of Public Health, University of São Paulo, São Paulo, Brazil; ^2^Center for Epidemiological Research in Nutrition and Health (NUPENS), University of São Paulo, São Paulo, Brazil; ^3^Faculty of Philosophy, Letters and Human Sciences, University of São Paulo, São Paulo, Brazil; ^4^Department of Nutrition, School of Public Health, University of São Paulo, São Paulo, Brazil

**Keywords:** availability, ultra-processed foods, community nutrition environment, consumer nutrition environment, spatial analysis

## Abstract

The availability of ultra-processed foods in a municipality tends to be related to different types of food retailers and their location in the territory, besides social, economic, and demographic factors. The aim of this study was to evaluate the availability of ultra-processed foods according to different types of food retailers and sociodemographic factors. This is a cross-sectional observational study carried out with audit data from food retailers in the municipality of Jundiaí, SP. Using a validated instrument, data on the availability of 18 types of ultra-processed foods were used to create a score of ultra-processed foods, ranging from 0 to 100 points, and five subscores referring to the ultra-processed food subgroups. Descriptive statistics and means comparison tests were performed to verify differences in the ultra-processed food availability score, according to the food retail type, household income, number of household members, and percentage of people of the color population in the census tract in which food retailers were located. Geo-referenced maps were used to characterize the score of ultra-processed in the territory, according to sociodemographic variables. A total of 649 food retailers were analyzed, most of which were classified as neighborhood markets (25.4%). The supermarkets were the category with the highest ultra-processed food availability score. Among the subgroups of ultra-processed foods analyzed, candies, soft drinks, and snacks were available in 60% of the food retailers. Higher ultra-processed food availability score was observed in regions with lower income, higher percentage of people of color population, and higher number of members per household. The findings show that the greater availability of ultra-processed foods is related to supermarkets and markets and regions of greater social vulnerability, which can put this population at nutritional risk.

## Introduction

Ultra-processed foods are formulations of ingredients, mostly of exclusive industrial use, that result from a series of industrial processes (“hence ultra-processed”) (1). Ultra-processed foods include carbonated soft drinks; sweet or savory packaged snacks; chocolate and candies (confectionery); ice cream; mass-produced packaged bread and buns; margarine and other spreads; cookies (biscuits), pastries, cakes, and cake mixes; breakfast “cereals”; preprepared pies and pasta and pizza dishes; poultry and fish “nuggets” and “sticks,” sausages, burgers, hot dogs, and other reconstituted meat products; powdered and packaged “instant” soups, noodles, and desserts; and many other products (1). These types of food products have become dominant in the food system (2), being easily found everywhere, often with attractive claims and aggressive marketing strategies, and placed on prominent shelves in several food retailers (3).

The sale of ultra-processed foods grows in almost every country in the world, especially among developing countries (4), and the massive presence of these foods generates an obesogenic environment (5–7). The high availability of these foods in food retailers is associated with greater consumption and low nutritional diet quality (8, 9). The strong relationship between the consumption of ultra-processed foods and an increased risk for obesity, diabetes, cancer, and mortality has been pointed out by systematic reviews and meta-analysis (10–12). As a result, it becomes increasingly important to study and monitor the availability of these foods in the food environment. In Brazil, data from the Household Budget Survey showed that the consumption of ultra-processed foods has been increasing as well as the correlation between high consumption of these foods and increased prevalence of several non-communicable diseases (NCD) as well as mortality (13).

Food availability is one of the key determinants of food choices in the consumer food environment (14). The consumer food environment has other relevant factors that interfere with food choices, such as price, quality of food, and positioning of products within a store (14–16). However, the availability of food is also conditioned to the categories of food retailers available in a region (17). In Brazil, there are different types of food retailers where the population purchases foods, such as supermarkets, grocery stores, convenience stores, bakeries, butchers, and fresh food markets (18).

In Brazil, some studies analyzed the food retailer type with the healthiness of the food purchased or consumed. A study conducted with data from the Household Budget Survey realized in 2008–2009 showed that ~50% of the Brazilian's food purchases are made at supermarket chains (16), and this action improves by 25% the chance of buying ultra-processed food (19). However, beyond supermarkets, a study using geospatial analysis with an ecological approach showed that there was a positive correlation between regular fruit and vegetable (FV) consumption and the density of FV specialized food markets (*r* = 0.497; *p* < 0.001) in the city of São Paulo-Brazil (20). Among the specialized FV food markets are the public supply markets that play an important role in access and availability of FV in Brazil and that need expansion to more socially vulnerable areas and technological renewal in face of the expansion of supermarket chains in recent years (20, 21).

Other countries, such as the United Kingdom and Mexico, also have high availability of unhealthy foods in the consumer food environment, with more peripheral and socially vulnerable neighborhoods having a higher density of establishments selling fast foods or ultra-processed foods (22, 23). The variation in the availability of healthy and unhealthy foods in the consumer food environment has been associated with different social, racial, and economic compositions (24–28).

The Dietary Guidelines for the Brazilian Population (29), which brings the Brazilian Government's official guidelines to promote healthy eating, as well as other international references (30–32), advise that, to have a healthy diet, it is necessary to adopt a diet based on fresh or minimally processed foods (mostly plant-based), sanitarily safe, in adequate amounts, and in accordance with the local food culture. Adequate access and availability of healthy foods in the food environment are essential for achieving nutritional recommendations. Purchasing food in places with a greater supply of fresh food and a lower supply of ultra-processed food favors the adoption of the recommendations (29).

The aim of this study was to evaluate the availability of ultra-processed foods, according to different types of food retailers and sociodemographic factors in a medium-sized municipality in Brazil.

## Methods

### Study Line

This is a descriptive cross-sectional study that was carried out with data obtained from an audit process of the consumer food environment in a medium-sized Brazilian municipality. The Research Ethics Committee of the School of Public Health of the University of São Paulo (protocol number 31019020.0.0000.5421) approved the study.

### Study Location

The data used in this study were collected in the municipality of Jundiaí, in the state of São Paulo. The municipality is located near the capital São Paulo, and it has 432.21 km^2^ of territorial area, with 97% of the urbanized area, and divided into 683 census sectors and 74 neighborhoods. It currently has 418,962 inhabitants (2020), being characterized as medium-sized, presenting a Human Development Index (HDI) of 0.82 and an average per capita income of R$ 1,121.82 (33, 34).

According to an estimate by the Brazilian Institute of Geography and Statistics in 2020, ~11% of Brazilian municipalities are considered medium-sized (50,000–100,000 inhabitants), among them the municipality of Jundiaí in the state of São Paulo. In this way, studying the food environment in a medium-sized municipality becomes relevant to the Brazilian scenario.

### Audit of Retail Stores

Data collection took place between the months of December 2017 and April 2018. Notably, 573 urban census tracts were surveyed, representing 83.9% of the total sectors in the municipality. Of the 16.1% unaudited census sectors, 66 were located in rural and environmental protection areas and 44 in neighborhoods with high violence rates as reported by local public managers. Every food retailer found on this route was audited, totaling 650 stores. For this study, only one store was excluded from the sample for lack of food availability data, totaling 649 food retailers analyzed. In a study carried out in the city of Jundiaí, SP in 2017 with secondary databases, a total of 960 food retailers were verified (35); however, a national study showed a low validity of secondary databases, requiring audits to better characterize the food environment (36).

The audit was carried out by six undergraduate students in nutrition from a private University in the municipality of Jundiaí, SP and three nutritionists worked as field supervisors. The researchers were trained in accordance with the “Application Manual for the Nutrition Environment Audit Instrument Based on the NOVA Food Classification of the Dietary Guidelines” protocol, developed especially to support field researchers during data collection (37). The instrument used in the audit process was AUDITNOVA (38), previously tested and validated, organized according to the NOVA food classification (39). It allowed capturing information such as retail type, food availability, price, promotions, brands, and advertising. From the variables available on AUDITNOVA, this study used data on the availability of 18 types of ultra-processed foods.

### Study Variables

To facilitate the analytical process, food retailers were grouped into 11 types based on the proposals of Costa et al. (40) and the Brazilian Institute of Geography and Statistics (IBGE) (41). which are as follows: (1) butcher/fishmonger, (2) public specialized fresh food market indoor, (3) private specialized fresh food market indoor, (4) neighborhood markets, (5) wholesaler/super/hyper/small chain market, (6) bakeries, (7) candies and snacks stores, (8) convenience stores, (9) beverage stores, (10) pharmacy and natural product stores, and (11) others (made up of pasta houses, dollar stores, and houseware stores).

For each category of food retail, availability data for the 18 types of ultra-processed foods were obtained. In addition, from this, a quantitative variable named ultra-processed food availability score was created, and it was standardized for a 100 scale. Thus, a food retailer that provides all 18 ultra-processed foods analyzed reaches a 100 score, and the one that does not sell any ultra-processed food scores 0 points. This score was developed specifically for this study in order to assess the concentrations of ultra-processed foods in the different types of food retailers in the territory. It should be noted that the 18 ultra-processed foods analyzed were the items that were audited with the AUDITNOVA instrument and that represent the most consumed ultra-processed foods by the Brazilian population according to the 2008–2009 Household Budget Survey (38, 42).

To further refine the analysis, the 18 ultra-processed food items were grouped into 5 subcategories according to the nutritional composition and reported in national studies on ultra-processed food consumption, namely (13) (1) sausages: hot-dog and pork sausage; (2) bakery products, cookies, and snacks: bread, breakfast cereals, snacks, and sandwich cookies; (3) sweets: ice cream, chocolate, and candy; (4) sugary beverages: soda can, 2-L soda, zero/light/diet soda, nectar, soft drink mix, and milk drink; (5) ready to eat foods: ready-made pizza, seasoning mix, and instant noodles.

The scoring of the subcategories was constructed based on the number of foods available in each and standardized to a scale of 100, for example, sausages–2 items (score 0–11); bakery products, cookies, and snacks–4 items (score 0–22); sweets–3 items (score 0–17); sugary beverages–6 items (score 0–33); and ready to eat foods–3 items (score 0–17).

To describe the score of ultra-processed food availability and the score of the ultra-processed food subcategory availability according to sociodemographic indicators, the following indicators extracted from the 2010 Demographic Census (by IBGE) were used: average monthly income of heads of household (classified as low/medium and high/very high); number of members per household (classified as 1–3 residents and more than 3 residents), and race or color (white, black, brown, indigenous, or yellow). For the race/color variable, we separated the white race/color from the others and created a variable with the participation of the people of the color population in the total of inhabitants (classified as up to 20% and >20%) (43). The chosen variables are often used as a proxy for poverty, quality of life, and to determine social inequalities in the country (44).

### Statistical Analyses

A descriptive analysis was performed on the food retailer type, ultra-processed food score, and subcategories based on the mean, standard deviation, median, and interquartile range (P25–P75).

The differences in the means of ultra-processed food scores according to sociodemographic indicators were analyzed using the Student's *t*-test, assuming significance values of *p* < 0.05. Stata14^®^ was used for statistical analysis.

To analyze the distribution of ultra-processed food availability scores in the territory, a two-stage spatial analysis was used. In stage 1, the geocoding process for all food retailers audited in the municipality was carried out using latitude and longitude coordinates. In stage 2, the interpolation of the ultra-processed food scores in the territory was performed, based on the score means by census tract. From points sampled in a given area, the method of Inverse Distance Weighting (IDW) interpolation uses the attribute estimates in unsampled locations. To estimate the value for some unsampled locations, the IDW will use the sampled values around this location, which will have a greater weight than the more distant values, assuming that things closer to each other are more similar than those more distant. Then, these variables were cross-checked with the indicators: number of members per household and participation of the people of the color population in the total of inhabitants. ArcGIS 10.0^®^ was used to develop the maps.

## Results

A total of 649 food retailers audited in the urban area of the municipality were evaluated in this study. The main food retailer types found were neighborhood markets 25.4% (*n* = 165), followed by pharmacies 18% (*n* = 117), and bakeries 14.5% (*n* = 94), and the public specialized fresh food market indoor appear less frequently (2.3%) in the municipality. The ultra-processed foods available at food retailers varied widely. The most available ultra-processed foods were candies, present in 77.8% of the food retailers analyzed, followed by soda cans, present in 72% of the food retailers, and 2-L sodas available in 67.5% of them. The food group ready-made pizzas (14.7%) and breakfast cereals (17.7%) were the least available foods (Table 1).

**Table 1:** Distribution (*n* and %) of food retailers and ultra-processed foods available at food retailers.

**Food retailers**	***n***	**%**
Neighborhood markets	165	25.4
Pharmacy, natural products	117	18.0
Bakeries	94	14.5
Candies and snacks stores	73	11.2
Convenience stores	49	7.5
Butchers/fishmongers	37	5.7
Wholesalers/super/hyper/small chain market	31	4.8
Beverage stores	26	4.0
Private specialized fresh food markets indoor	23	3.5
Others	19	2.9
Public specialized fresh food markets indoor	15	2.3
**Ultra-processed foods available at food retailers**		
Ready-made pizza	95	14.6
Breakfast cereals	114	17.6
Milk drinks	142	21.9
Pork Sausage	183	28.2
Seasoning mix	211	32.5
Hot-dog	217	33.4
Sliced bread	242	37.3
Nectar	288	44.4
Instant noodles	295	45.4
Soft drink mix	330	50.8
Ice cream	346	53.3
Sandwich cookies	347	53.6
Zero/light/diet soda	383	59.0
Chocolate	384	59.2
Chips	399	61.5
Soda 2 liters	438	67.5
Soda can	465	71.6
Candy	504	77.7

*Jundiaí, SP, Brazil, 2017–2018*.

The average ultra-processed food availability score, counted for each type of food retailer, was higher in supermarkets (mean score = 93.4), neighborhood markets (mean score = 75.0), and bakeries (mean score = 55.8). The lowest ultra-processed food availability score was found in public specialized fresh food market indoor (mean score = 8.5). When we analyzed the median and interquartile values (p25 and p75), we observed great variations in the ultra-processed food availability score even within the same type of food retailers, such as butchers and fishmongers (p25 = 16.7; p75 = 61.1). The same is observed in the categories of public and private specialized fresh food market indoor, in which we observed a median equal to 0 for ultra-processed food availability score; however, within the same category, we have retailers with a score higher than 70.0 (Table 2).

**Table 2:** Mean (SD), median (p25, p75) of the ultra-processed availability score according to the food retailer type.

	**Ultra-processed availability score**			
**Food retailers**	**Mean score (SD)**	**Median score**	**P25**	**P75**
Neighborhood markets	75.0 (21.9)	77.8	61.1	94.4
Pharmacy, natural products	13.4 (10.1)	11.1	5.5	16.7
Bakeries	55.8 (15.9)	55.5	44.4	66.7
Candies and snacks stores	22.8 (20.3)	16.7	5.5	38.9
Convenience stores	48.9 (15.0)	50.0	38.9	61.1
Butchers/fishmongers	38.0 (25.4)	33.3	16.7	61.1
Supermarkets	93.4 (17.2)	100.0	94.4	100.0
Beverage stores	21.8 (14.5)	27.8	11.1	33.3
Private specialized fresh food markets indoor	43.5 (36.3)	0.0	38.9	77.8
Others	34.5 (16.2)	27.8	22.2	50.0
Public specialized fresh food markets indoor	8.5 (22.0)	0.0	0.0	5.5

*Jundiaí, SP, Brazil, 2017–2018*.

*Among the 649 establishments audited, 70 of them did not have any ultra-processed food items, having water or in natura foods as a priority sale*.

**Figure 1:**
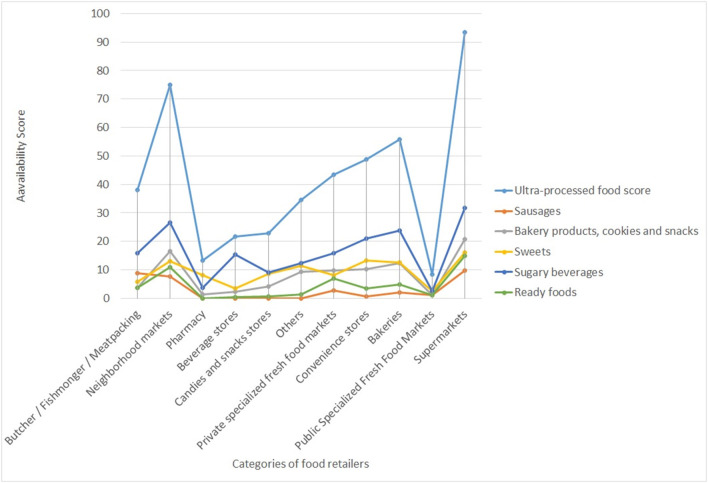
Mean score of the ultra-processed food subcategories availability according to different types of food retailers. Jundiaí, SP, Brazil, 2017–2018.

The subcategories of ultra-processed foods made it possible to identify which type of food has the greatest contribution to the total score of ultra-processed foods within the categories of food retailers. It can be observed that 9 out of the 11 food retailers evaluated had higher average scores for the availability of sugary beverages than for other subgroups of ultra-processed foods. Another food category that was highly available in the food retailers was sweets, followed by bakery products, cookies, and snacks (Figure 1).

Statistical differences in the mean score of the ultra-processed food availability were observed in food retailers located in lower-income regions [mean (SD) = 53.6 (30.8)] and higher percentage of people of the color population [mean (SD) = 54.3 (30.5)] (*p* < 0.001). For the subcategories of ultra-processed foods studied, it was possible to observe that all subgroups presented statistically higher mean scores in low and middle-income regions, in households with 3+ members, and in regions above 20% participation of people of color population. The only subgroup of ultra-processed foods for which it was not possible to observe differences in the mean scores according to sociodemographic factors was the sweets subgroup (Table 3).

**Table 3:** Ultra-processed food availability score and subcategories, according to sociodemographic variables.

**Sociodemographic Variables**	**Ultra-processed food availability score**	**Score of the ultra-processed food subcategories availability**				
		**Sausages**	**Bakery products, cookies and snacks**	**Sweets**	**Sugary beverages**	**Ready to eat foods**
	**Mean (SD)**	**Mean (SD)**	**Mean (SD)**	**Mean (SD)**	**Mean (SD)**	**Mean (SD)**
**Head of household average income**						
Low to medium income	53.6 (30.8)^*^	4.3 (5.1)^*^	11.5 (8.1)^*^	10.8 (4.9)	20.4 (11.3)^*^	6.6 (6.3)^*^
High/very high income	41.1 (31.5)	2.8 (4.5)	8.1 (8.0)	10.4 (5.6)	15.6 (12.1)	4.1 (5.9)
**Number of members per household**						
1–3 residents	38.6 (1.7)^*^	2.2 (0.2)^*^	7.4 (0.4)^*^	10.7 (0.3)	14.7 (0.7)^*^	3.5 (0.3)^*^
>3 residents	52.9 (1.7)	4.5 (0.3)	11.3 (0.4)	10.4 (0.3)	20.1 (0.6)	6.6 (0.3)
**Participation of black, brown, yellow and indigenous population in total inhab**.						
Up to 20%	41.8 (31.6)^*^	2.8 (4.5)^*^	8.3 (8.1)^*^	10.5 (5.6)	15.8 (12.0)^*^	4.3 (6.0)^*^
>20%	54.3 (30.5)	4.5 (5.2)	11.6 (8.1)	10.7 (4.8)	20.7 (11.3)	6.7 (6.2)

*Jundiaí, SP, Brazil, 2017–2018*.

* *Significant mean difference on Student's t-test at level p ≤ 0.001*.

**Figure 2:**
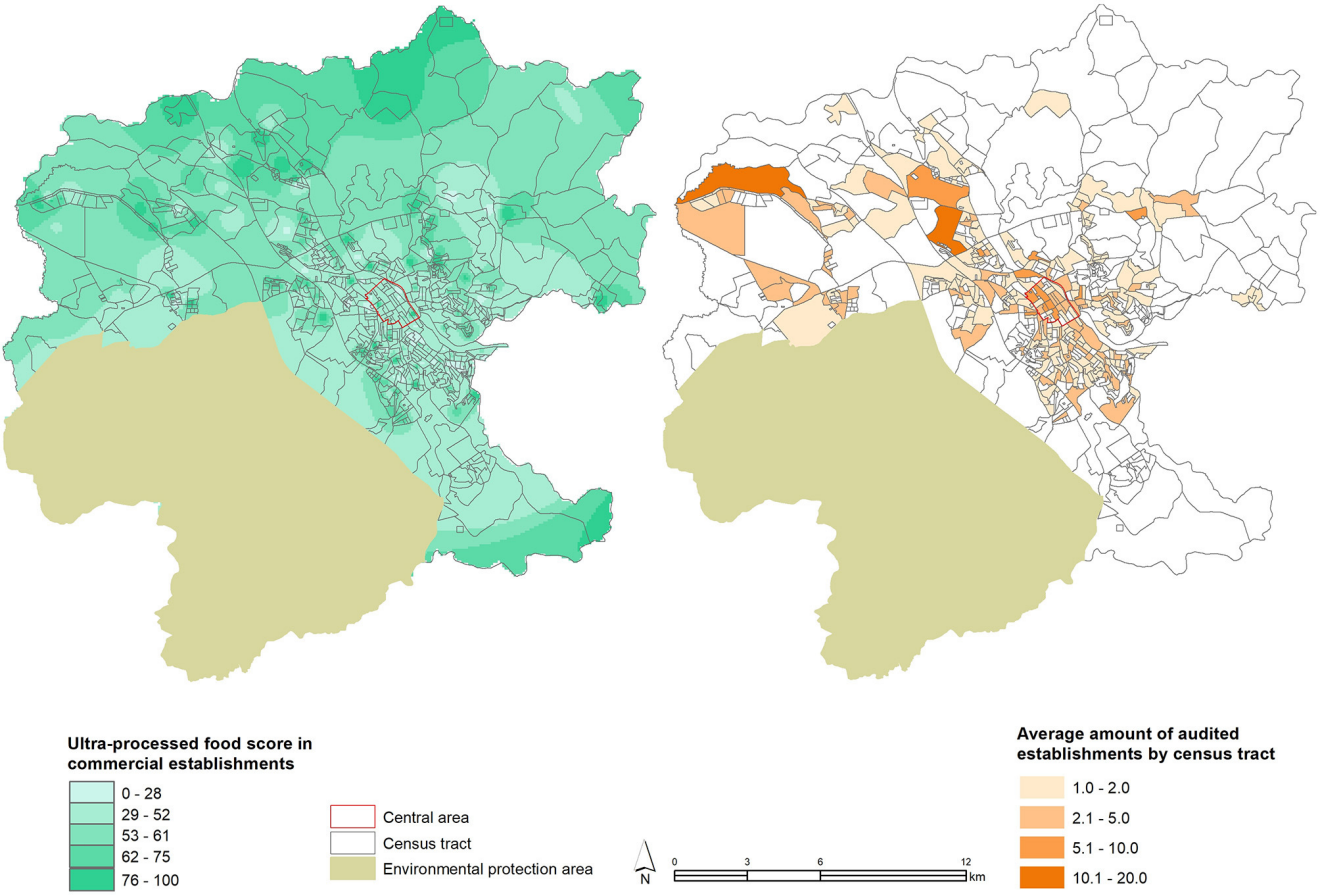
Ultra-processed food availability score and number of establishments audited in the census tract of the municipality of Jundiaí, SP, Brazil, 2017–2018.

Figure 2 illustrates the characterization of the ultra-processed food availability score on the municipality map, as well as the average number of food retailers audited in the urban territory. The maps reveal that food retailers with higher scores are found in the peripheral regions of the municipality, and some points are dispersed in the central area. There are census tracts in the peripheral areas of the municipality with a low number of food retailers, which are mostly concentrated around the central region, leaving many peripheral sectors without food retailers.

**Figure 3:**
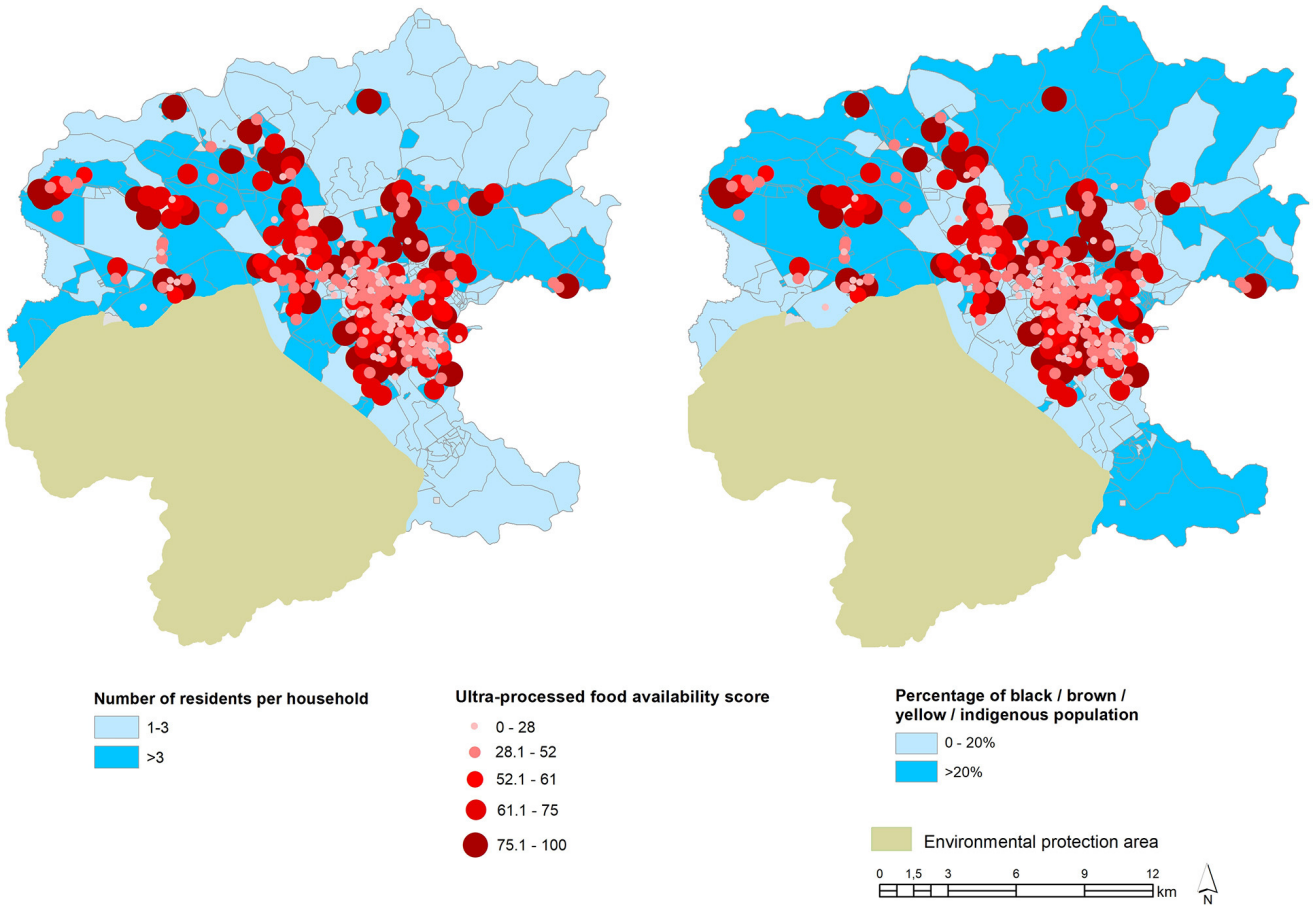
Ultra-processed food availability score and sociodemographic variables in the municipality of Jundiaí, SP, Brazil, 2017–2018.

Figure 3 illustrates the ultra-processed food availability score and the sociodemographic variables. In the central, neighborhoods are concentrated in the households with the largest number of members (3+) and the highest scores of ultra-processed foods. In the peripheral regions, we observed the largest number of residents being people of color (above 20%) and many sectors with a total absence of commercial establishments or with high scores of ultra-processed foods.

## Discussion

This study aimed to evaluate the availability of ultra-processed foods, according to different types of food retailers and sociodemographic factors in a medium-sized municipality in Brazil. To evaluate these issues, an ultra-processed food availability score was developed and applied to food retailers. In the municipality studied, supermarkets, neighborhood markets, bakeries, and convenience stores had the highest scores of ultra-processed food availability. This study improved the understanding of socio-demographic factors related to the availability of ultra-processed foods in the territory and found that neighborhoods with a higher concentration of people with low and middle income, a higher number of residents per household, and a higher percentage of people of color were associated with food retailers with the highest scores of ultra-processed foods. Mapping the score across the territory of the municipality made it possible to observe regions characterized as food deserts or food swamps according to the literature (45).

The choice of creating a score to assess the availability of ultra-processed foods in the consumer food environment came from another study carried out in Brazil by Borges et al. (46), which developed and tested a score to assess the healthiness of the consumer food environment considering factors such as food availability, price, advertising, and placement strategies in the final score composition. When testing the healthiness score in a sample of food retailers, the authors found that butchers and fishmongers and public specialized fresh food market indoor have the best healthiness indexes and are the places that could favor the best food choices. Other results that are similar to the findings of this study are that supermarkets and convenience stores had the worst healthiness scores, which also took into account the high availability of ultra-processed foods.

The great availability of the food retailers that sell most unhealthy foods associated with the low availability of healthy foods has been seen as a risk factor for the obesity pandemic in several countries worldwide, especially in deprived regions (6, 25, 27). In our study, it was possible to observe that in the socioeconomically disadvantaged areas, there are less food retailers available, and when they are available, they present a high score for the availability of ultra-processed foods, unlike the central areas of the city where there is a greater diversity of food retailers and with a lower score for ultra-processed foods. In Brazil, other studies have also found that the most peripheral neighborhoods have difficult access to markets that sell fresh and healthy food and are more likely to have zones of food deserts or food swamps (47). Some hypotheses may explain this phenomenon in the most peripheral areas, among them: the unequal amount of public supply equipment (48) that ensures access to healthy foods such as food street markets, farmers markets, community gardens, and grocery stores that are usually located in more central areas of cities (26); lower-income communities had limited economic access to healthy food compared with the wealthier ones (22, 49). Also, some studies have indicated that poorer neighborhoods had worse economic access to healthy food (50, 51).

Food price can also be an important factor that hinders access to healthy foods and facilitates the consumption of ultra-processed foods. In Brazil, a study showed that fresh foods (such as meat, milk, fruits, and vegetables) tend to cost more than ultra-processed foods (52). The very characteristics of ultra-processed foods such as hypertaste (due to high amounts of sugar, salt, and fat in its composition), aggressive marketing, convenience, and practicality (ready to eat anywhere and without the need for culinary preparation with elaborate techniques) can also contribute to the widespread presence of these foods in most retail stores and their frequent consumption by the Brazilian population (39, 42).

In this study, it was possible to assess the consumer food environment from the current healthy eating perspective adopted by the Dietary Guidelines for the Brazilian Population (29). According to national recommendations, healthy eating should be based on fresh and minimally processed foods and culinary preparations based on these foods and should avoid the consumption of ultra-processed foods. In this sense, our findings, as observed in other studies (8, 53, 54), confirm that public specialized fresh food market indoor had the lowest scores of ultra-processed foods and may be good options for the population to achieve the nutritional recommendations. However, the population still faces obstacles in achieving a healthy diet, especially regarding the supply and availability of food in food retailers.

Regarding that, the study showed that the highest score of ultra-processed food availability was found in supermarkets. Machado et al. (19) showed that shopping in supermarkets increases the purchase of ultra-processed foods by 25% in Brazil. The same trend was also observed in international studies showing that the greater availability of ultra-processed foods at supermarkets is a determining factor for the consumption of these foods (45, 55–59). In this study, almost all the subcategories of ultra-processed foods were available in supermarkets and neighborhood markets, and almost all were more prevalent in lower-income regions. However, the sweet food group was the only one that was not associated with the sociodemographic factors studied. Our hypothesis for this finding is that it is a group of food so widely spread in all types of food retailers present in neighborhoods of all social classes and that it was not possible to note statistical differences.

The sugary beverages are the most available ultra-processed foods among all types of food retailers (around 70% of them have them available), even more at supermarkets. A study in the United States found that sugary beverages accounted for 40% of household spending compared with all drinks purchased (45). In the city of São Paulo, a greater variety of sugary beverages was related to a higher prevalence of consumption, especially when the point of sale of these drinks is close to the residence (9). The high availability of sugary beverages in the consumer food environment increases the chance of consuming these products, which has been associated, in particular, with a high prevalence of diabetes mellitus (5, 60). Therefore, the discussion of taxing sugary beverages has been conducted by different countries as a regulatory action to build healthier food environments and to discourage purchases. Mexico, Chile, and South Africa have already implemented this policy and observed a reduction in purchases and consumption of these products after the implementation of the policy (61).

Although supermarkets and neighborhood markets have high availability of ultra-processed foods, another retail group that was highly prevalent in the municipality and that attracted our attention was pharmacies. The pharmacies represent 18% of the retailers in the municipality. The decision to include this retailer group in the analysis was because this type of retail, in addition to selling medicines, are also allowed to sell food items called convenience items such as water, milk drinks, candies, mints, and products with functional purposes such as diet and light, according to the health rules of the state of São Paulo, Brazil. It is observed that most foods sold in pharmacies are classified as ultra-processed foods (62, 63). In contrast, pharmacies limit themselves to commercialize some subtypes of ultra-processed foods, especially candies and snacks and sugary beverages, which is why the score found for them ended up being low (average score = 13) when compared with that observed for supermarkets (average score = 93). In pharmacies, these foods are located on check-out lines, and several studies show that food sale in these areas influences impulse buying (64, 65).

It was identified in this study that the greater availability of ultra-processed foods in the food retailers was related to the socio-demographic conditions of the region in which they are located, exposing them to a situation of greater food vulnerability to those who live or purchase food in these regions. In an international study that evaluated the relationship between the healthiness of food retailers with sociodemographic factors, it was shown that living in a low-income neighborhood with a higher percentage of black population exposes this population to lower availability of healthy foods and poorer diet quality (59). Other studies show that economically vulnerable populations have worse access to food when compared with individuals living in more economically favored neighborhoods (66, 67).

As for the ethnic aspects and the greater concentration of establishments selling ultra-processed foods in these neighborhoods, it brings up a reflection on the social, economic, political, and health inequalities historically present in Brazilian society and that affect, in particular, the poor and black people. In the United States, studies that explore ethnicity and the food environment observe that blacks and Latinos tend to live in areas with a lower density of healthy establishments, which reflects the segregation condition in which these populations live (68). Our study reflects the characteristic of the Brazilian population in which mixed races often have less income and education than whites and are segregated in peripheral neighborhoods with poor infrastructure (69) and, as seen in this study, with high availability of ultra-processed foods in the territory.

These data show the importance of developing and implementing public policies for the food environment that consider the social, economic, and racial inequalities to which the Brazilian population is exposed. The main policies that are recommended to protect individuals and guide them to make healthier choices are as follows: taxing sugary drinks, reducing the price of healthy foods, expanding public supply markets and community gardens, and regulating the advertising of food around schools (30, 70).

The concept of food deserts and swamps emerges in the literature to identify differences in access to healthy foods according to different socioeconomic indicators (71, 72). Identifying food deserts and swamps in a municipality with a high human development index, such as Jundiaí, SP, Brazil, is a reflection of the complexity of developing actions that guarantee the human right to adequate and healthy food for all. In line with this study, a survey carried out in the municipality of Juiz de Fora, located in the state of Minas Gerais, Brazil, which also has a high human development index, observed that regions with greater social vulnerability had a lower density of food retailers (73).

Geospatial analyses provided a broad view of the territory regarding the characterization of the food environment in the community beyond the access to the consumer food environment and are very important to understand inequalities in food access. It makes it possible to identify regions with low food retailer availability or where the only food purchase options had a high score for ultra-processed foods.

This study opens a window of opportunity to discuss local food supply policies and how necessary they are to make access to healthy food less unequal in the territory. Moreover, the discussion should also be made in the sense of further promoting food retailers that have lower scores for ultra-processed foods, such as butchers and fishmongers, and public or private specialized fresh food market indoor in order to facilitate healthy food choices by the population. It is worth mentioning that the United Nations 11th Sustainable Development Goal addresses the aspect of sustainable cities and communities. In this sense, managers should discuss aspects that make the urban environment healthier and more sustainable, and the discussion of food retail and food supply should be a priority in this aspect, in addition to traffic policies, air pollution, and among other aspects frequently discussed (74). The methodology created in this study and the results presented could support the discussion of urban space as a promoter of healthy eating or not.

Among the strengths of this study is the use of the AUDITNOVA instrument (38) for data collection, which made it possible to analyze the consumer food environment from the perspective of the ultra-processed foods (39) and dialogue with the recommendations of the Dietary Guidelines for the Brazilian Population. The wide variety of food retailers present in this study was also a positive aspect because it allowed checking variations in the score of ultra-processed foods according to the different types of food retailers. The crossing of data from the audit process of the consumer food environment with the sociodemographic variables available in the 2010 census (IBGE) also allowed a broader look at the inequality of food access in the municipality. Another strong point of this study was the scope of the audits, ~84% of the census tracts were covered with the collection of data from food retailers throughout this territory.

Among the limitations of this study, there is the fact that the availability of healthy foods (fresh and minimally processed) has not been explored. In contrast, the availability of ultra-processed foods is supported by data in the literature proving that the impact of ultra-processed foods on health does not depend on the availability of healthy foods (18, 59). A second limitation would be the limited external validity of the study that evaluates just one Brazilian municipality. The construction of an expanded panorama of the Brazilian consumer food environment might be explored by future studies with sampling from different locations, using a comparable methodology. However, the audit took place in more than 80% of the studied territory, so we believe that it is possible to extrapolate these data to other medium-sized municipalities in Brazil with similar characteristics. We have not been able to assess other socioeconomic indicators that can also interfere with food choices, such as access to safe water for consumption and meal preparation, food prices, marketing strategies, and food policies. We also did not map other possible sources/places where people from peripheral regions acquire their food: food baskets, food banks, NGOs, community centers, popular restaurants, community gardens, among other possibilities.

In this coronavirus disease 2019 (COVID-19) pandemic, the protective measures implemented to contain the spread of contamination had a direct impact on the retail sector on the ways food is sold and consumed. We observed an increase in food insecurity in the country, uncertainty about food prices, uncertainty about minimum food stocks, and about the availability of food items due to hygienic-sanitary conditions (75). Social distancing brought a physical barrier to food access, access to equipment such as street markets, gardens, and small producers was restricted in several Brazilian municipalities, and shopping conditions were reduced to supermarket-type establishments that offered safe solutions or online shopping (76). In this pandemic moment, mapping the food environment and identifying food availability becomes of great importance in order to identify and map emergency supply policies that can guarantee access to healthy foods, especially for the most socially vulnerable (75, 77, 78). This period has shown us how necessary it is to create stable strategies or public supply policies that guarantee access to healthy foods without the population being held hostage by the private sector, which provides high amounts of ultra-processed foods in the poorest neighborhoods, as shown in this study.

In conclusion, it was observed that, at the local level, the availability of ultra-processed foods varies according to the category of food retailer and the sociodemographic configurations of the territories. The findings show that the greater availability of ultra-processed foods is related to supermarkets and neighborhood markets and regions of greater social vulnerability, which can put this population at nutritional risk. Considering the several factors that influence the construction of the consumer food environment and the various ways that it impacts the food choices of individuals, the local characterization of the availability of ultra-processed foods has the potential to expand initiatives to foster food environments that promote health and nutrition and to amplify the discussions about the sustainable urban environment.

## Data Availability Statement

The original contributions presented in the study are included in the article/supplementary material, further inquiries can be directed to the corresponding author/s.

## Ethics Statement

The study was approved by the Research Ethics Committee of the School of Public Health under number 31019020.0.0000.5421. Written informed consent for participation was not required for this study in accordance with the national legislation and the institutional requirements.

## Author Contributions

PS and CB: conceptualization. PS: methodology, data curation, and writing—original draft preparation. PS, CB, and WC-M: formal analysis. PS, CB, WC-M, and PJ: writing—review and editing. PJ: supervision. All authors have read and agreed to the published version of the manuscript.

## Conflict of Interest

The authors declare that the research was conducted in the absence of any commercial or financial relationships that could be construed as a potential conflict of interest.

## Publisher's Note

All claims expressed in this article are solely those of the authors and do not necessarily represent those of their affiliated organizations, or those of the publisher, the editors and the reviewers. Any product that may be evaluated in this article, or claim that may be made by its manufacturer, is not guaranteed or endorsed by the publisher.
